# Integrated media and plant growth regulators comparative evaluation for enhanced *in vitro* propagation and acclimatization of *Selenicereus Costaricensis*

**DOI:** 10.1186/s12870-026-08246-x

**Published:** 2026-02-17

**Authors:** Eman Z. Ahmed, Eman T. Hussien, Mohamed F. Ahmed, Mohamed A. Nasser, Ashraf B. Abdel Razik, Hebatallah Aly

**Affiliations:** 1 Botany Department, Faculty of Science, Capital University, Cairo, 11795 Egypt; 2 Dry and Saline Farming Technology Department, Arid Land Agricultural Graduate Studies and Research Institute, Ain shams University, 68-Hadayek Shoubra, 11241 Cairo, Egypt; 3 Department of Horticulture, faculty of agriculture, Ain Shams University, 68-Hadayek Shoubra, Cairo, 11241 Egypt; 4 Department of Genetic, faculty of agriculture, Ain Shams University, 68-Hadayek Shoubra, Cairo, 11241 Egypt

**Keywords:** Dragon fruit, Pitaya, *In vitro* culture, MS, WPM, BAP, Morphophysiological description

## Abstract

**Background:**

several protocols were tested for in vitro propagation of dragon fruit *Selenicereus costaricensis* (F.A.C. Weber) S.Arias & N. Korotkova ex Hammel, but face limitations such as low multiplication rates, slow growth, and poor rooting and acclimatization. Media composition affects morphophysiological properties and dragon plant growth throughout all propagation stages. This study presents a comprehensive and optimized protocol for in vitro micropropagation and *ex vitro* acclimatization of *S. costaricensis*, addressing these challenges. Tip explants from two-week-old seedlings were cultured on Murashige and Skoog (MS) and Woody Plant Medium (WPM) at varying strengths (¼, ½, ¾, and full) and sucrose concentrations (20 and 30 g/L).

**Results:**

The optimal shoot proliferation was attained on ¾-strength MS medium supplemented with 30 g/L sucrose, yielding shoots with an average length of 1.9 ± 0.0003 cm and a fresh weight of 1.36 ± 0.057 g per plantlet. The multiplication of ¾ MS supplemented with 2 mg/L BAP and 0.5 mg/L kinetin yielded the best proliferation rate, producing 70.66 ± 0.577 shoots per explant. Rooting was markedly improved on ¼ MS with activated charcoal, yielding roots averaging 13.6 ± 0.0004 cm in length and 21 ± 0.0004 roots per plantlet. During acclimation, plantlets cultivated in a peat moss: perlite (1:1) mixture attained a 97 ± 1.000% survival rate, produced 3.6 ± 0.577 shoots per plantlet, and developed vigorous root systems. .

**Conclusion:**

To optimize in vitro propagation of dragon fruit plantlets, use ¾ MS medium with 30 g/L sucrose for establishment, add 2 mg/L BAP for multiplication, then transfer axillary shoots to ¼ MS with activated charcoal for rooting, and acclimatize using a 1:1 peat moss–perlite mix.

## Background

The agricultural field is currently facing the dual difficulties of abiotic and biotic stresses and an economic crisis, which threaten horticultural and ornamental production [1–5]. However, within these concerns, opportunities are emerging for the renewal of plant material. The pitaya (dragon) fruit exhibits considerable promise as a novel agricultural commodity for Mediterranean cultivators due to its low water requirements and remarkable adaptability to the elevated temperatures commonly encountered within greenhouse environments [6, 7]. *S. costaricensis*, known as the red pitaya, Costa Rican pitahaya, or Costa Rica night-blooming cactus, is a cactus species native to Costa Rica, Panama, and Nicaragua. The species is a significant crop in the family Cactaceae and is grown commercially for its fruit [8, 9]. It is an economically and nutritionally viable fruit crop in dry climates and regions with water shortages, making it a sustainable option for cultivation in arid areas [10]. Furthermore, there is a growing market demand for novel and nutritious exotic fruits, and these products are globally recognized and valued as superfruits [6].

This impressive plant boasts large, bright magenta flowers with a scallop-lobed stem, ovate to globose fruit, and pear-shaped seeds. An easily cultivated, fast-growing epiphyte or xerophyte. The plant is resilient, adapting well to challenging environments [11]. Red dragon fruits are rich in nutrients such as vitamin C, minerals, antioxidants, and various biologically active constituents [12–14]. The fruit is consumed fresh or used in various food products. The utilization of dragon fruit peels is widely reported as being rich in anthocyanin, pectin, and antioxidants [15–17].

The propagation of dragon fruit often occurs through the utilization of seeds or cuttings [18, 19]. However, these approaches are insufficient due to slow growth of seeds, the limited supply of young nodes, and genetic variability [20]. Therefore, rapid, high-quality, and efficient micropropagation techniques are essential for improved production. Plant tissue culture serves as a viable alternative technique to produce plantlets on a large scale [21, 22]. While various investigations have been conducted on the general propagation techniques of dragon fruit, there is currently a lack of comprehensive knowledge regarding the standardized procedures for generating superior planting material for dragon fruit through tissue culture methods. Several studies of [23–25] have proposed procedures for the micropropagation of dragon fruit. Axillary multiplication and bioreactor culture were more efficient and reliable techniques for the mass propagation of pitaya [26]. The impact of phytohormones (Benzylaminopurine and Naphthalene acetic acid) on callus formation, shoot, and root regeneration was documented [27–29]. However, these publications do not provide a thorough understanding of the main factors involved in the large-scale production of dragon fruit planting materials.

Dragon fruit micropropagation has been the subject of numerous studies, although the majority have concentrated on certain phases, such as shoot regeneration or roots, which has led to protocols with limited rates of multiplication, weak root systems, or poor acclimation performance. Furthermore, basal media strengths, sucrose levels, and cytokinin/auxin combinations for *S. costaricensis* have not been thoroughly compared in prior research, nor have physiological responses been incorporated into protocol optimization. Thus, a thorough, stage-specific assessment that determines the best circumstances from establishment to acclimation is still clearly needed [20].

Several studies have demonstrated that supplementing the culture medium with benzylaminopurine (BAP), a synthetic cytokinin, significantly enhances shoot initiation and multiplication in various plant species. BAP promotes cell division and axillary bud activation, thereby breaking apical dominance and encouraging multiple shoot formation. the presence of BAP, in combination with varying sucrose concentrations, leads to a substantial increase in shoot number [30]. Reported that BAP was a more suitable growth hormone for maximum shoot bud differentiation and multiple [31–33]. Several studies have shown that enhancing the growing medium with benzylaminopurine (BAP) has a positive effect on shoot initiation and multiplication [34]. Similarly, in *Maxillaria picta*, BAP supplementation at 9 µM significantly improved shoot and leaf formation during in vitro culture, although higher concentrations led to tissue browning and reduced photosynthetic pigment accumulation [35].

The addition of activated charcoal (AC) to the rooting medium has been widely recognized as a beneficial practice in plant tissue culture due to its multifaceted role in improving morphophysiological responses. AC possesses a highly porous structure with an extensive surface area, enabling it to absorb inhibitory compounds such as phenolic exudates, toxic metabolites, and excess plant growth regulators that often accumulate in culture media and hinder rhizogenesis. By removing these substances, AC creates a more favorable environment for root initiation and elongation. Furthermore, AC can absorb ethylene and abscisic acid, both of which negatively affect root formation, while simultaneously reducing oxidative stress and tissue browning, thereby enhancing explant vigor and survival rates [36, 37]. In addition to its detoxifying effect, AC provides a darkened environment that mimics natural soil conditions, which is known to stimulate root induction. Recent studies have also indicated that AC may gradually release adsorbed nutrients and growth-promoting substances, contributing to improved photosynthetic pigment content and overall plantlet performance during the rooting stage [32].

This study was conducted to develop and optimize a comprehensive in vitro micropropagation and *ex vitro* acclimatization protocol for *S. costaricensis* (red dragon fruit) by evaluating the effects of media type, strength, sucrose concentration, plant growth regulators, and acclimatization substrates to enhance shoot multiplication, rooting efficiency, and plantlet survival so that more plantlets with healthy shoot and root systems can be produced to meet the demand of increasing commercial cultivation.

## Materials and methods

### Materials

Tissue culture media (M0222) Murashige and Skoog medium (MS) was purchased from Duchefa Biochemie, and WPP03 Woody Plant Medium (WPM) was purchased from Cassion Laboratories. Phytohormones were purchased from Sigma.

### Culture conditions

Media salts and sucrose were dissolved in distilled water with a magnetic stirrer, and pH was adjusted to 5.8 using KOH and HCl. The medium was solidified with 6 g/L agar, melted at 90 °C, and dispensed (35–40 mL) into 400 mL glass jars with polypropylene lids. Sterilization was performed at 121 °C and 100 kPa for 15 min. Media were stored at 25 ± 3 °C for up to five days before use. Cultures were incubated at 24 ± 2 °C under a 16 h light/8 h dark photoperiod (2000–2500 lx) using LED lighting.

### Plant materials

The tip explants from two-week-old seedlings of dragon fruits (*S. costaricensis*, red pericarp, reddish-purple flesh) were provided from Vitro Lab (for plant tissue culture work, Masr-Alex desert road, Egypt). The seedlings were produced from seeds that were sterilized and cultured on MS [38] in jar vessels with 5 seeds per jar. The media was supplemented with 30 g/L sucrose and solidified with 6 g/L agar.

### Shoot culture establishment

The tip explants pieces 1–2 cm in length cultured on two different types of in vitro plant growth regulators -free culture media, MS and WPM [39], with different media strengths (¼, ½, ¾, and full-strength), and two concentrations of sucrose (20 g/L and 30 g/L) for each media type. The treatments are a combination of the previous variables, as listed in Table 1. Plant growth regulators -free basal media were used as the zero- plant growth regulators controls for all establishment treatments and comparisons.

**Table 1:** Treatments used in the study for the culture establishment

Medium	Sucrose concentration g/L	Treatment (Media strength, media type, and sucrose concentration)			
MS	20	¼ MS1	½ MS1	¾ MS1	MS1
	30	¼ MS2	½ MS2	¾ MS2	MS2
WPM	20	¼WPM1	½WPM1	¾WPM1	WPM1
	30	¼WPM2	½WPM2	¾WPM2	WPM2

### Shoot multiplication

After 4 weeks, stem cuttings were used as explant material. Based on the results of the previous experimental stage, two media types (¾ MS2 and ¾ WPM2) were selected for multiplication. The media was supplemented with kinetin (Kin) 0.5 mg/L combined with different concentrations of benzyl aminopurine (BAP) (2, 2.5, and 3 mg/L). Treatments are presented in Table 2.

**Table 2:** Treatments for the multiplication media of *S. costaricensis*

Treatment	Media composition
¾ MS2-BAP1	¾ MS + 30 g Sucrose + 2 mg/L BAP + 0.5 mg/L Kin
¾ MS2-BAP2	¾ MS + 30 g Sucrose + 2.5 mg/L BAP + 0.5 mg/L Kin
¾ MS2-BAP3	¾ MS + 30 g Sucrose + 3 mg/L BAP + 0.5 mg/L Kin
¾ WPM2-BAP1	¾ WPM + 30 g Sucrose + 2 mg/L BAP + 0.5 mg/L Kin
¾ WPM2-BAP2	¾ WPM + 30 g Sucrose + 2.5 mg/L BAP + 0.5 mg/L Kin
¾ WPM2-BAP3	¾ WPM + 30 g Sucrose + 3 mg/L BAP + 0.5 mg/L Kin

### Rooting stage

For rooting of axillary shoots (4 weeks old), ¼ MS or ¼WPM were tested with or without activated charcoal (AC). All media were supplemented with 0.1 mg/L indole butyric acid (IBA), 0.5 mg/L Kin, and 15 g/L sucrose. Rooting media treatments are listed in Table 3 as follows.

**Table 3:** Treatments of rooting media supplemented with Indole Butyric acid (IBA), Kinetin (Kin), and with or without activated charcoal (AC)

Treatment	Media composition
¼ MS	¼ MS + 15 g/L Sucrose + 0.1 ml/L IBA + 0.5 ml/L Kin
¼ WPM	¼ WPM + 15 g/L Sucrose + 0.1 ml/L IBA + 0.5 ml/L Kin
¼ MS-AC	¼ MS + 15 g/L Sucrose + 0.1 ml/L IBA + 0.5 ml/L Kin + 0.5 g/L AC
¼ WPM-AC	¼ WPM + 15 g/L sucrose + 0.5 g/L + 0.1 ml/L IBA + 0.5 ml/L Kin + 0.5 g/L AC

### Acclimatization stage

Four-week-old plantlets from ¼ MS + AC, the optimal rooting treatment, were removed from culture jars and carefully cleaned under running water to remove any residual media. After a 30-minute soak in a fungicide mixture (Rezolex 1 g/L), the plantlets were potted and acclimatized for 25 days in a greenhouse (32 ± 5 °C, 60–70% relative humidity) using either peat moss: perlite (1:1) or peat moss: vermiculite (1:1) to determine the best acclimatization conditions. Plantlet survival percentage, height, shoot number, root length, root number, and plantlet fresh weight/plantlet during *ex vitro* acclimatization stage of dragon were estimated.

### Morphological and physiological parameters

The morphological and physiological characteristics were assessed for the stages of multiplication, rooting, and acclimation. This facilitates the selection of optimal settings at each stage.

The identified morphological parameters for each stage were as follows: Shoot length (SL) and fresh weight (FW) were assessed during the establishment phase; SL, shoot count (the number of branched for a plantlet), and FW were measured during the multiplication stage; SL, root length (RL), root count, and plantlet FW were evaluated during the rooting phase; After 25 days of acclimatization, $\:Survival\:Rate\:\left(\%\right)=\frac{Number\:of\:survived\:plantlets}{Total\:number\:of\:plantlets}X100$, plantlet height, shoot count, root count, RL, and plantlet FW were recorded. The physiological parameters measured were chlorophyll a (Chl a), Chl b, carotenoids, total pigments, soluble sugars, proteins, phenolics, and flavonoids. These variable metabolites were estimated as follows: The total pigments was estimated according to the protocol of Metzener et al. [40] by using 10 mL of 80% acetone, 1 g of each sample was extracted from fresh, completely expanded adult leaves. The absorbance of the extract was measured spectrophotometrically at wavelengths of 660 nm, 640 nm, and 440 nm for Chl a, Chl b, and carotenoids, respectively; the total soluble sugars were estimated according to Umbreit et al. [41]; the total proteins were estimated according to Bradford [42] fresh plant tissue samples were homogenized in chilled phosphate buffer (pH 7.0) and centrifuged at 10,000 × g for 15 min at 4 °C to obtain a clear supernatant. Aliquots of 100 µL from the extract were mixed with 5 mL of Bradford reagent (Coomassie Brilliant Blue G-250) and incubated at room temperature for 5 min to allow color development. Absorbance was measured at 595 nm using a UV–Vis spectrophotometer. Protein concentration was determined by comparing the absorbance values against a standard calibration curve prepared using bovine serum albumin (BSA) in the same extraction buffer. All measurements were performed in triplicate to ensure accuracy; total phenolic contents were determined according to the method described by Kujala et al. [43] using Folin–Ciocalteu reagent and gallic acid as a standard. It was expressed as mg gallic acid equivalents (GAE) per g dry tissue (mg GAE/g dry weight of extract); total flavonoid was estimated using aluminum chloride colorimetric assay according to [44, 45], and the content was expressed in milligrams quercetin equivalents (QE) per gram extract.

### Statistical analysis

A completely randomized experimental design was performed. Each treatment comprised ten replicates, with each replicate representing a container that held five explants. Data were analyzed employing one-way ANOVA for each growth stage, and treatment means were distinguished using Tukey’s HSD test at *P* ≤ 0.05. Analyses were conducted utilizing Minitab 19, whereas correlation matrices and heatmaps were produced employing R (metan package). Assumptions of ANOVA, including normality and homogeneity of variance, were confirmed prior to conducting the analysis.

## Results

### Shoot culture establishment

**Fig. 1:**
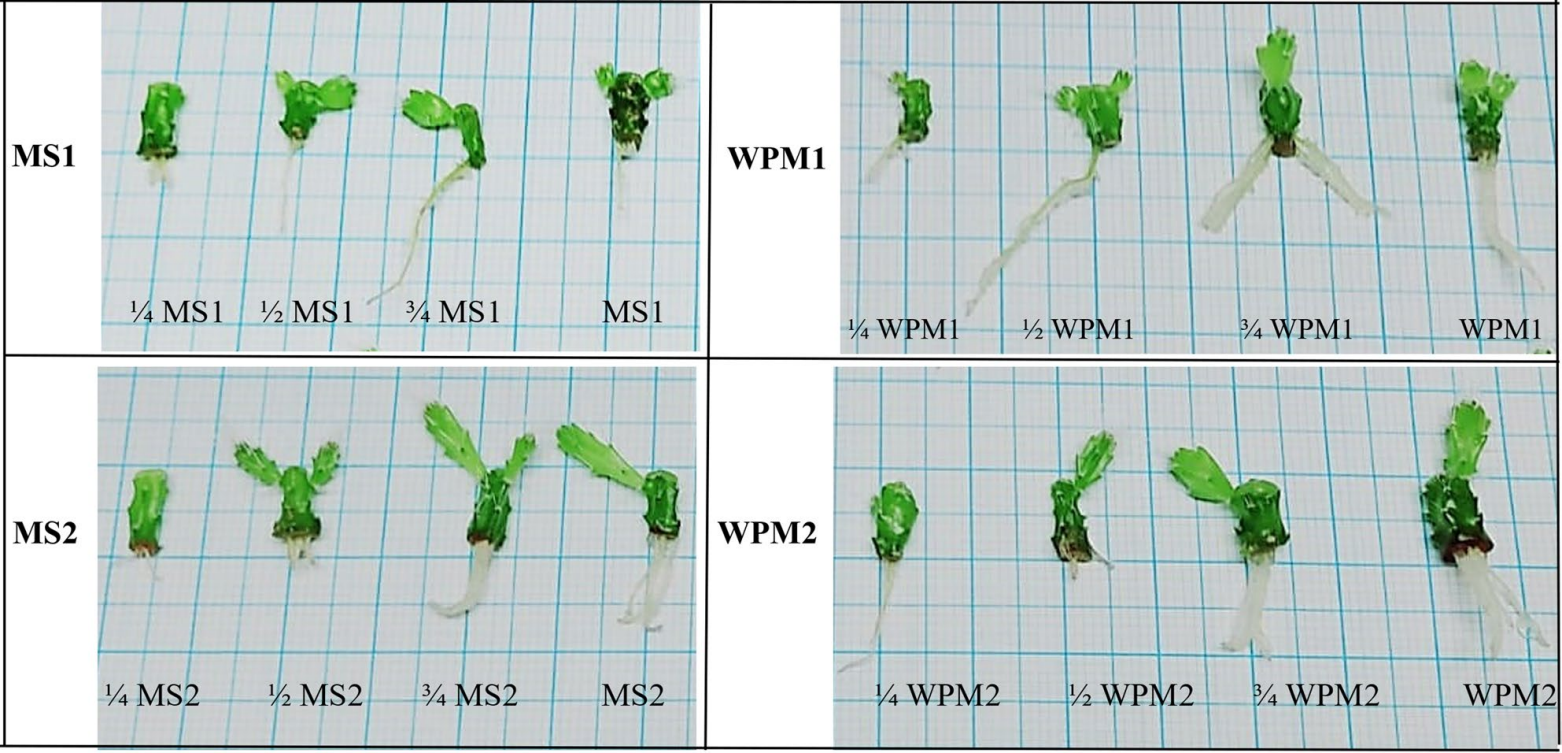
Phenotypes of *S. costaricensis* in response to the variable culture media types and compositions

**Fig. 2:**
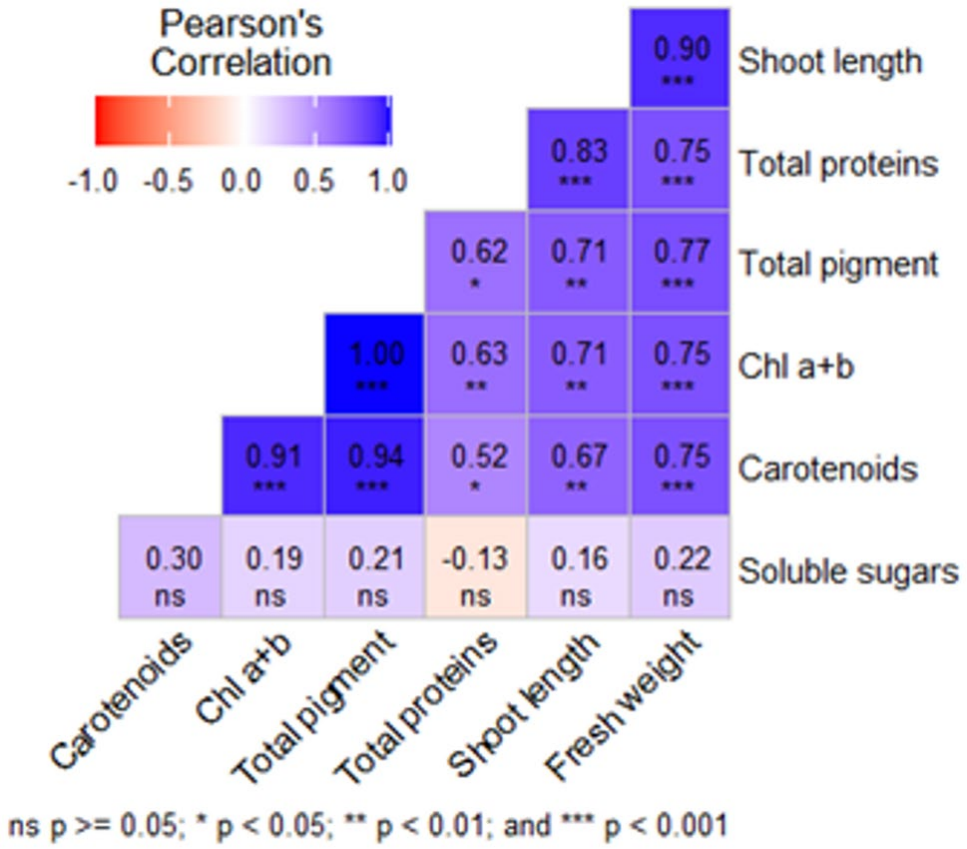
Correlations among the morphological and physiological parameters of *S. costaricensis* in the establishment stage

Concerning morphological traits, different treatments significantly influenced dragon shoot length and fresh weight and were positively correlated (Table 4; Fig. 1). The treatments ¾ MS2 and ¾ WPM2 were optimal for SL with 1.9 and 1.66 cm, respectively, and FW with 1.366 and 1.00 g/plant, respectively. The WPM2 medium showed comparable results with 1.36 cm SL and 0.96 g FW. Concerning physiological Traits, all the physiological parameters in the establishment stage showed highly significant differences among all the treatments (Table 5). The concentration of total photosynthetic pigments (Chla + b, and carotenoids) was highest in shoots cultured on ¾ MS2 (0.303 mg/g FW) followed by shoots cultured on ¾ WPM2 (0.159 mg/g FW). Both ¾ MS2 and WPM2 significantly accumulated total proteins with 22.3 mg/g DW. The correlation among the morphological and physiological parameters is positive, except for soluble sugars with total proteins, which had a negative correlation as shown in Fig. 2. The correlation was strong, *P* < 0.5, between shoot length and fresh weight with total protein and total pigments, and weak, *P* ≥ 0.5, with soluble sugars.

**Table 4:** Means of physiological traits of *S. costaricensis* explants during establishment in response to media type, media strength, and sucrose concentration

Treatment	Shoot length (cm)	Fresh weight (g)
¼ MS1	0.76 ± 0.057 h	0.400 ± 0.000 g
½ MS1	1.26 ± 0.057de	0.593 ± 0.011de
¾ MS1	1.366 ± 0.057^cd^	0.743 ± 0.011^c^
MS1	1.066 ± 0.057^fg^	0.796 ± 0.005^c^
¼ MS2	1.00 ± 0.000^g^	0.543 ± 0.005^ef^
½ MS2	1.33 ± 0.057^cde^	0.596 ± 0.005^de^
¾ MS2	1.90 ± 0.000^a^	1.366 ± 0.057^a^
MS2	1.46 ± 0.057^c^	0.966 ± 0.057^b^
¼WPM1	0.83 ± 0.057^h^	0.246 ± 0.005^h^
½WPM1	1.00 ± 0.000^g^	0.416 ± 0.005^g^
¾WPM1	1.300 ± 0.000^de^	0.496 ± 0.005^f^
WPM1	1.200 ± 0.000^ef^	0.656 ± 0.005^d^
¼WPM2	1.066 ± 0.057^fg^	0.430 ± 0.017^g^
½WPM2	1.000 ± 0.000^g^	0.513 ± 0.005^f^
¾WPM2	1.66 ± 0.057^b^	1.000 ± 0.000^b^
WPM2	1.66 ± 0.115^b^	0.956 ± 0.005^b^

Different letters in each column indicate significant differences at *P* ≤ 0.05

MS1 = Murashige and Skoog medium with 20 g/L sucrose,MS2 = Murashige and Skoog medium with 30 g/L sucrose

WPM1 = Woody Plant Medium with 20 g/L sucrose,WPM2 = Woody Plant Medium with 30 g/L sucrose

**Table 5:** Means of physiological traits of *S. costaricensis* explants during establishment in response to media type, media strength, and sucrose concentration

Treatment	Chl a + b (mg/g FW)	Carotenoids (mg/g FW)	Total pigment (mg/g FW)	Soluble sugars (mg/g DW)	Total proteins (mg/g DW)
¼ MS1	0.09 ± 0.0009^cd^	0.011 ± 0.0001 ^d^	0.101 ± 0.0010^k^	82.40 ± 61.30^c^	16.52 ± 0.373^c^
½ MS1	0.11 ± 0.0010^bc^	0.021 ± 0.0003^bc^	0.131 ± 0.0015^fg^	102.71 ± 0.446^bc^	17.58 ± 0.302^bc^
¾ MS1	0.12 ± 0.0015^b^	0.023 ± 0.0003^b^	0.143 ± 0.0010^d^	116.26 ± 0.535^abc^	19.45 ± 0.387^b^
MS1	0.11 ± 0.0010^bc^	0.026 ± 0.0003^b^	0.135 ± 0.0010^ef^	158.45 ± 0.425^a^	14.50 ± 0.452^cd^
¼ MS2	0.10 ± 0.0010^bc^	0.018 ± 0.0002^c^	0.118 ± 0.0010^i^	108.26 ± 0.384^bc^	12.40 ± 0.487^cd^
½ MS2	0.11 ± 0.0010^bc^	0.021 ± 0.0003^bc^	0.131 ± 0.0005^fg^	97.533 ± 0.503^bc^	18.56 ± 0.445^bc^
¾ MS2	0.25 ± 0.0022^a^	0.052 ± 0.0006^a^	0.303 ± 0.0020^a^	116.67 ± 0.516^abc^	22.28 ± 0.544^a^
MS2	0.09 ± 0.0010^c^	0.021 ± 0.0003^bc^	0.111 ± 0.0010^j^	107.75 ± 0.486^bc^	18.77 ± 0.370^bc^
¼ WPM1	0.06 ± 0.0009^cd^	0.007 ± 0.0001^e^	0.067 ± 0.0015^l^	84.433 ± 0.513^c^	13.72 ± 0.563^cd^
½ WPM1	0.11 ± 0.0010^bc^	0.026 ± 0.0004^b^	0.136 ± 0.0015^e^	112.58 ± 0.688^abc^	14.56 ± 0.420^cd^
¾ WPM1	0.11 ± 0.0010^bc^	0.020 ± 0.0003^bc^	0.130 ± 0.0005^g^	140.51 ± 0.415^ab^	17.59 ± 0.525^bc^
WPM1	0.10 ± 0.0010^bc^	0.015 ± 0.0002^cd^	0.115 ± 0.0015^ij^	135.76 ± 1.570^ab^	15.25 ± 0.477^c^
¼ WPM2	0.11 ± 0.0010^bc^	0.016 ± 0.0002^cd^	0.126 ± 0.0015^h^	128.56 ± 0.513^abc^	12.55 ± 0.506^cd^
½ WPM2	0.13 ± 0.0015^b^	0.018 ± 0.0002^c^	0.147 ± 0.0011^c^	108.66 ± 1.528^bc^	16.85 ± 0.257^c^
¾ WPM2	0.14 ± 0.0015^b^	0.019 ± 0.0002^c^	0.159 ± 0.0010^b^	109.56 ± 0.513^bc^	22.33 ± 0.550^a^
WPM2	0.12 ± 0.0015^b^	0.021 ± 0.0003^bc^	0.142 ± 0.0015^d^	106.14 ± 0.307^bc^	18.73 ± 0.643^bc^

Different letters in each column indicate significant differences at *P* ≤ 0.05

MS1 = Murashige and Skoog medium with 20 g/L sucrose, MS2 = Murashige and Skoog medium with 30 g/L Sucrose

WPM1 = Woody Plant Medium with 20 g/L sucrose, WPM2 = Woody Plant Medium with 30 g/L sucrose

### Shoot multiplication

**Fig. 3:**
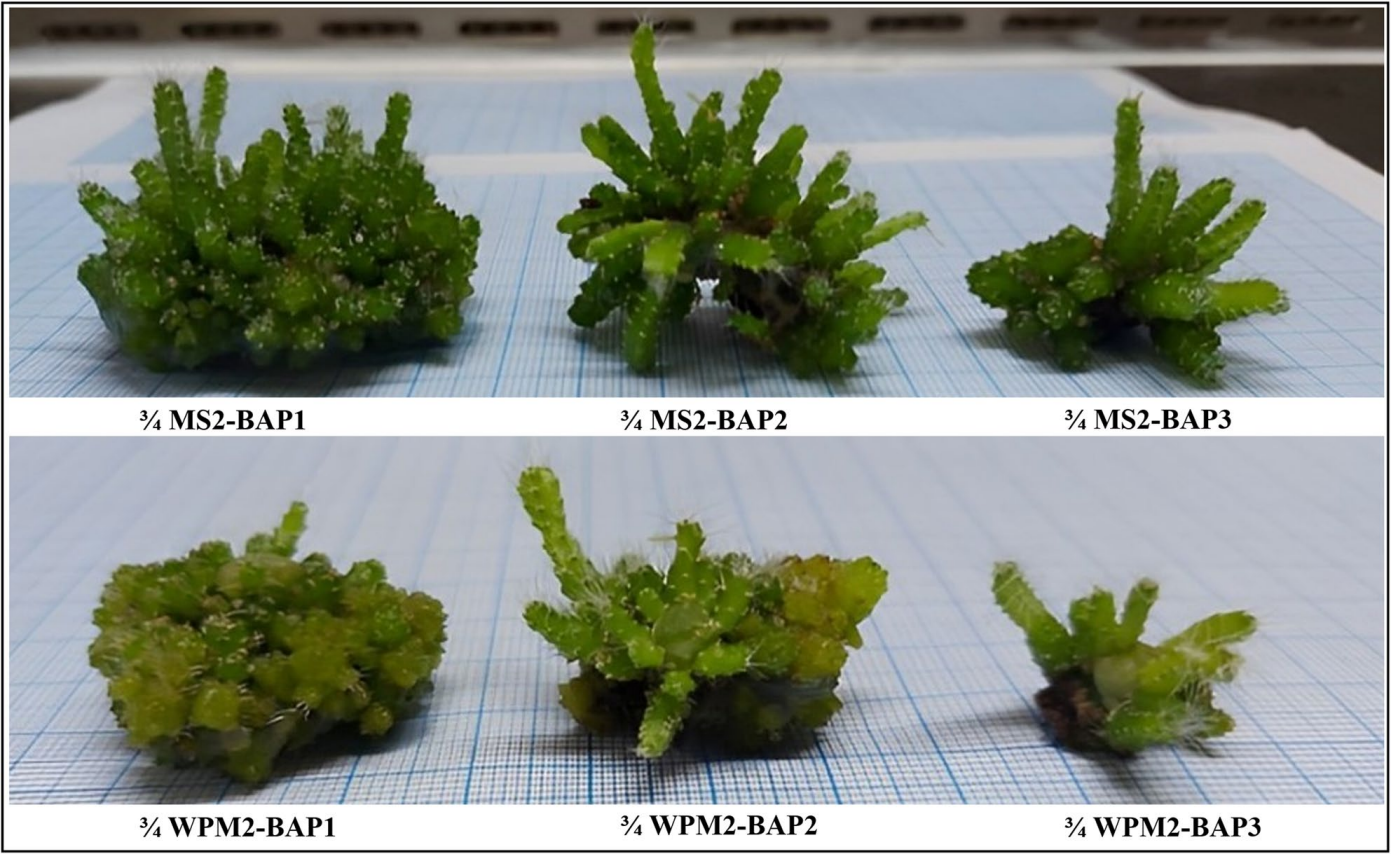
Phenotypes of *S. costaricensis* in response to the multiplication culture media

**Fig. 4:**
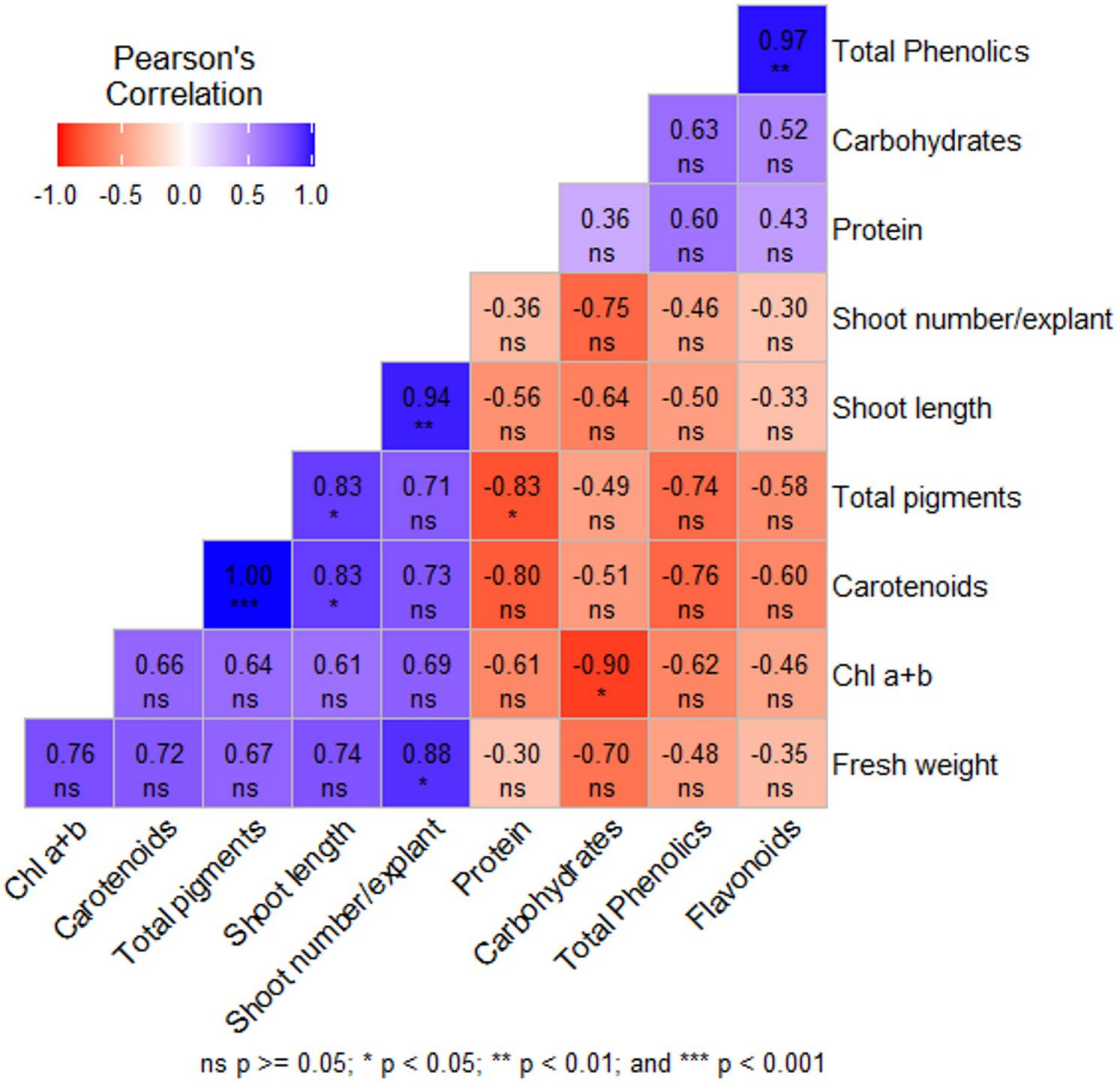
Correlations among the morphological and physiological parameters of *S. costaricensis* in the multiplication stage

Concerning morphological Traits, After selecting the best media strength in the establishment stage, plant growth regulators concentration of BAP (2, 2.5 and 3 mg/L) and 0.5 mg/L kin were applied to the selected treatments for multiplication stage. The ¾ MS2-BAP1 media showed the highest shoot number (70.66 shoot/explant) and fresh weight, followed by ¾ MS2-BAP2 (Table 6). The highest shoot length was detected in ¾ MS2-BAP2, followed by ¾ MS2-BAP1. Regarding the media type, ¾MS2 was better in clustering formation, number of shoots, shoot length, and more regular shoot formation (Fig. 3). Concerning Physiological Traits, media type (MS or WPM) significantly affects the pigment formation as in Table 7. The correlation among the morphological and physiological parameters represented by Fig. 4 showed that There was a negative correlation between the number and length of shoots with flavonoids, total phenolics, carbohydrates and proteins, and a positive correlation with chloride A + B, carotenoids, and total pigments. the photosynthetic pigments strongly correlated with each other, total phenolics and flavonoids are also strongly related. Different pigments (Chl a + b, carotenoids, and total pigments) negatively correlated with carbohydrates, total proteins, phenolics, and flavonoids.

**Table 6:** Morphological parameters of *S. costaricensis* in the multiplication stage as affected by two media types, each media supplemented with different concentrations of benzyl aminopurine

Treatment	Shoot length (cm)	Shoot number/explant	Fresh weight (g)
¾ MS2-BAP1	2.900 ± 0.000 ^b^	70.66 ± 0.577 ^a^	10.41 ± 0.000 ^a^
¾ MS2-BAP2	3.066 ± 0.057 ^a^	64.00 ± 0.000 ^b^	7.660 ± 0.000 ^b^
¾ MS2-BAP3	2.100 ± 0.000 ^c^	21.66 ± 0.577 ^c^	3.916 ± 0.005 ^d^
¾ WPM2-BAP1	0.900 ± 0.000 ^f^	3.000 ± 0.000 ^f^	4.786 ± 0.005 ^c^
¾ WPM2-BAP2	1.700 ± 0.000 ^d^	7.000 ± 0.000 ^e^	3.413 ± 0.005 ^e^
¾ WPM2-BAP3	1.500 ± 0.000 ^e^	11.00 ± 0.000 ^d^	1.310 ± 0.000 ^f^

Different letters in each column indicate significant differences at *P* ≤ 0.05

MS2 = Murashige and Skoog medium with 30 g/L sucrose, WPM2 = Woody Plant Medium with 30 g/L sucrose

BAP1 = 2 mg/L benzyl aminopurine, BAP2 = 2.5 mg/L benzyl aminopurine, BAP3 = 3 mg/L benzyl aminopurine.

**Table 7:** Physiological parameters of *S. costaricensis* in the multiplication stage as affected by two media types, each media supplemented with different concentrations of benzyl aminopurine

Treatment	Chl a + b (mg/g FW)	Carotenoids (mg/g FW)	Total pigments (mg/g FW)	Carbohydrates (mg/g DW)	Protein (mg/g DW)	Total Phenolics (mg gallic acid equivalents /g DW)	Flavonoids (mg quercetin equivalents /g DW)
¾ MS2-BAP1	0.143 ± 0.001 ^a^	0.079 ± 0.001 ^b^	0.22 ± 0.0006^a^	37.58 ± 0.456^e^	137.1 ± 0.551^c^	52.08 ± 0.556^e^	5.15 ± 0.257^d^
¾ MS2-BAP2	0.142 ± 0.001 ^ab^	0.070 ± 0.002 ^b^	0.21 ± 0.0006^a^	47.06 ± 0.595^d^	109.0 ± 1.530^e^	87.78 ± 0.525^c^	14.4 ± 0.503^a^
¾ MS2-BAP3	0.141 ± 0.001 ^b^	0.070 ± 0.001 ^a^	0.21 ± 0.006^c^	45.89 ± 2.670^d^	90.60 ± 0.503^f^	50.78 ± 0.525^f^	5.12 ± 0.261^d^
¾ WPM2-BAP1	0.125 ± 0.001 ^c^	0.035 ± 0.001 ^d^	0.13 ± 0.006^a^	61.84 ± 0.537^c^	158.1 ± 0.608^b^	93.4 ± 0.721^b^	13.3 ± 0.513^ab^
¾ WPM2-BAP2	0.093 ± 0.001 ^d^	0.061 ± 0.001 ^c^	0.19 ± 0.003^c^	88.36 ± 0.705^a^	128.2 ± 0.643^d^	84.78 ± 0.525^d^	11.7 ± 0.513^c^
¾ WPM2-BAP3	0.091 ± 0.001 ^e^	0.028 ± 0.001 ^e^	0.12 ± 0.006^b^	67.59 ± 1.357^b^	176.8 ± 1.010^a^	95.09 ± 0.605^a^	12.9 ± 0.551^bc^

Different letters in each column indicate significant differences at *P* ≤ 0.05

MS2 = Murashige and Skoog medium with 30 g/L sucrose, WPM2 = Woody Plant Medium with 30 g/L sucrose

BAP1= 2 mg/L benzyl aminopurine, BAP2= 2.5 mg/L benzyl aminopurine, BAP3= 3 mg/L benzyl aminopurine

### Rooting stage

**Fig. 5:**
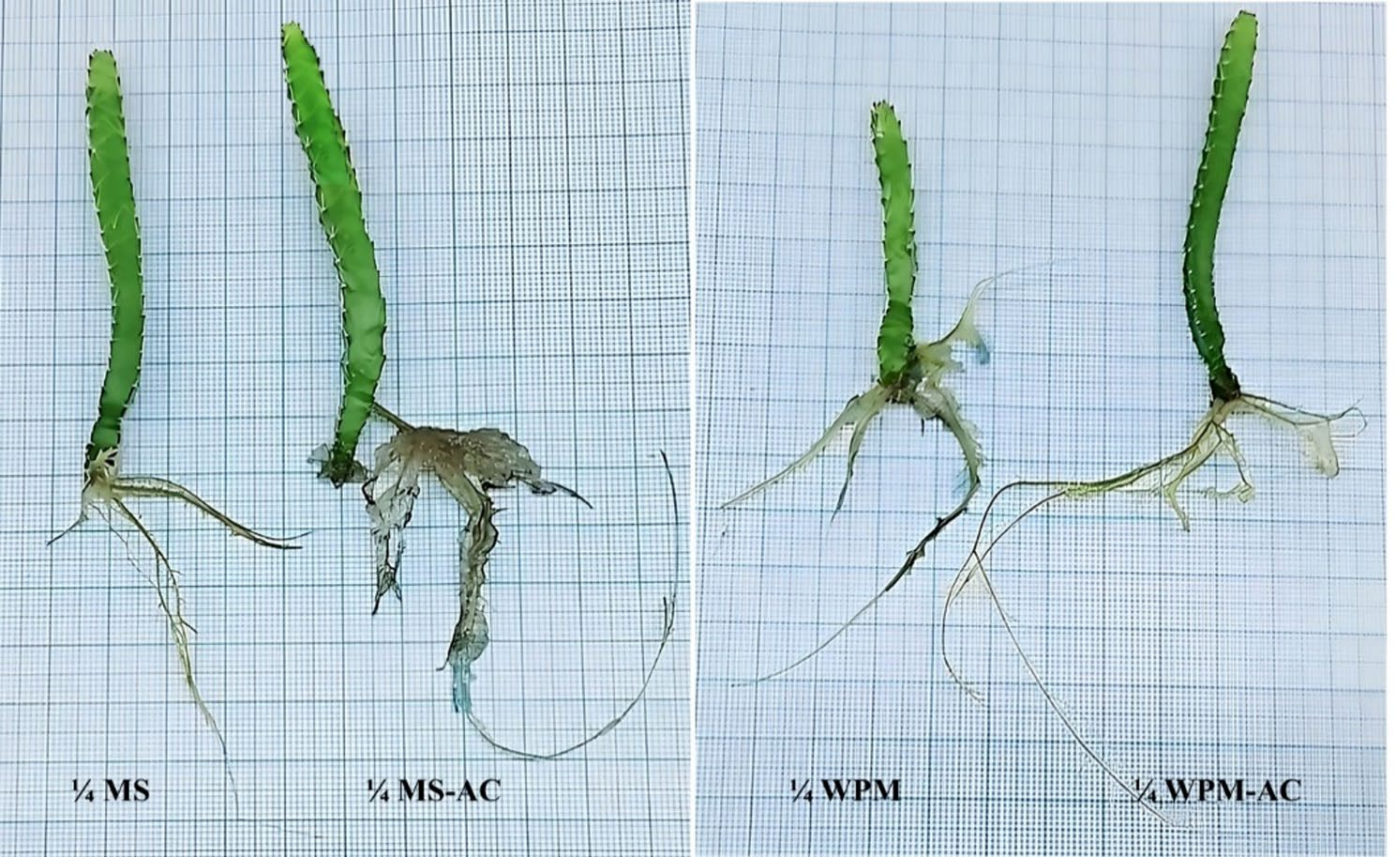
Phenotypes of *S. costaricensis* in response to the rooting culture media

**Fig. 6:**
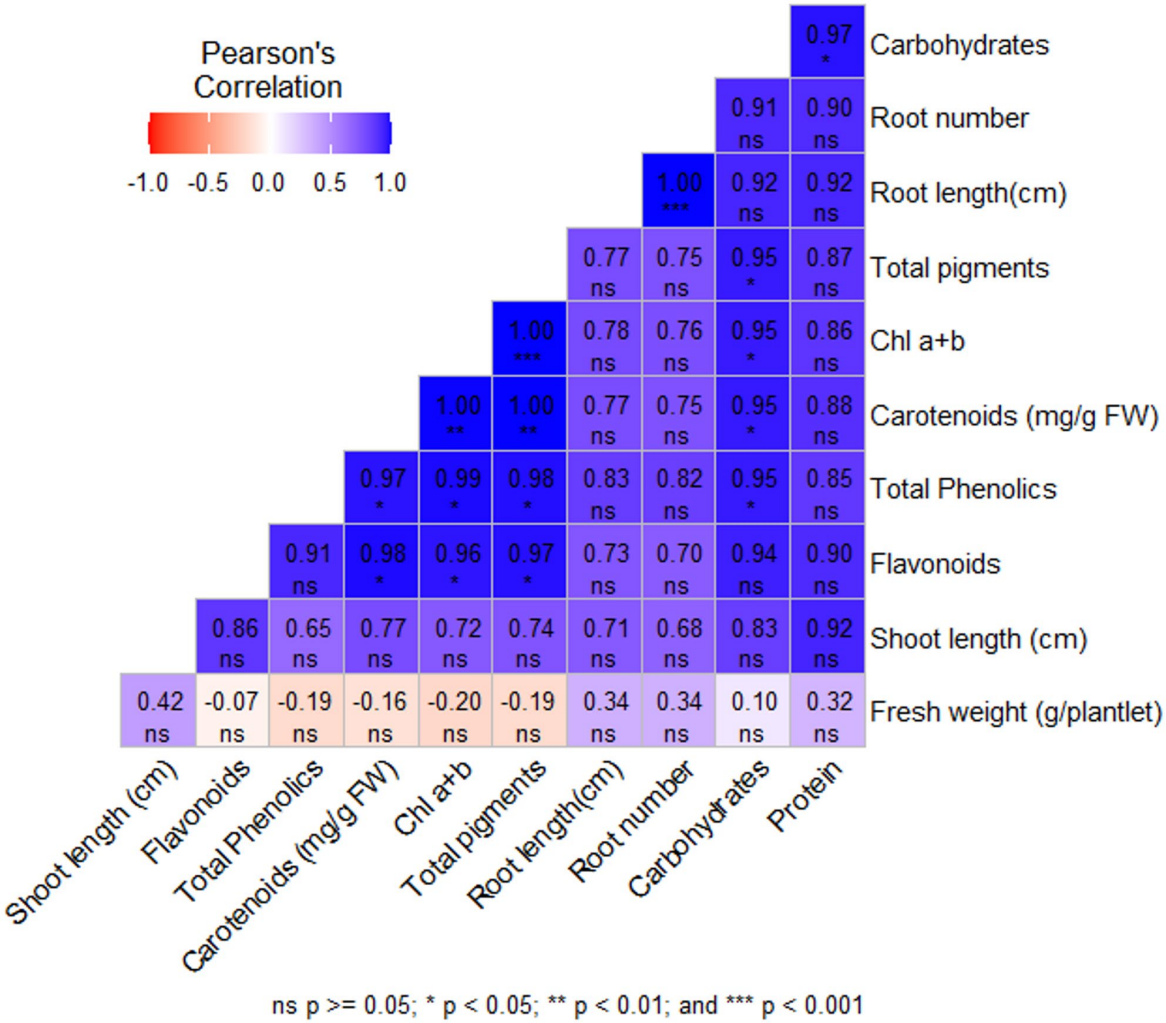
Pearson’s correlations among the morphological and physiological parameters in the rooting stage of *S. costaricensis*

Concerning morphological Traits, In rooting stage, ¼ media strength of MS and WPM was used, without or with activated charcoal (AC). The addition of AC enhanced the root length and numbers in both ¼ MS and ¼ WPM; MS media with AC was the best (Table 8). Shoot growth was also improved in the presence of AC (Fig. 5). Concerning physiological Traits, At the physiological level, there is a significant difference among all the physiological parameters. The ¼ MS-AC significantly promoted the accumulation of photosynthetic pigments, carbohydrates, total proteins, phenolics, and flavonoids followed by ¼ WPM-AC (Table 9). The correlations among the morphological and physiological parameters in (Fig. 6) showed positive relations among all the parameters, particularly between root length and number. The variables were positively correlated as shown in Fig. 6.

**Table 8:** Means of shoot and root lengths, root number, and plantlet fresh weight of *S. costaricensis* after rooting. Two media were used, each without or with charcoal (AC)

Treatment	Shoot length (cm)	Root length(cm)	Root number	Fresh weight (g/plantlet)
¼ MS	7.500 ± 0.000^c^	7.900 ± 0.000^c^	9.000 ± 0.000^c^	1.720 ± 0.000^b^
¼ WPM	5.566 ± 0.057^d^	7.566 ± 0.057^d^	9.000 ± 0.000^c^	1.430 ± 0.000^c^
¼ MS-AC	7.900 ± 0.000^a^	13.60 ± 0.000^a^	21.00 ± 0.000^a^	2.226 ± 0.057^a^
¼ WPM-AC	7.700 ± 0.000^b^	12.70 ± 0.000^b^	19.00 ± 0.000^b^	1.250 ± 0.000^d^

Different letters in each column indicate significant differences at *P* ≤ 0.05

**Table 9:** Physiological parameters of *S. costaricensis* at the rooting stage. Two media were used (¼ MS and ¼ WPM), each without or with charcoal (AC)

Treatment	Chl a + b (mg/g FW)	Carotenoids (mg/g FW)	Total pigments (mg/g FW)	Carbohydrates (mg/g DW)	Protein (mg/g DW)	Total Phenolics (mg/g DW)	Flavonoids (mg/g DW)
¼ MS	0.301 ± 0.001 ^c^	0.088 ± 0.001^c^	0.39 ± 0.010c	87.14 ± 0.622^c^	100.7 ± 0.808^b^	50.31 ± 1.076^cd^	6.85 ± 0.539^bc^
¼ WPM	0.273 ± 0.001 ^d^	0.067 ± 0.002^d^	0.34 ± 0.015^d^	51.53 ± 0.500^d^	81.2 ± 0.643^c^	49.05 ± 0.563^de^	5.69 ± 0.512^c^
¼ WPM-AC	0.323 ± 0.001 ^b^	0.098 ± 0.002^b^	0.42 ± 0.010^b^	139.53 ± 0.500^b^	120.5 ± 1.61^a^	54.24 ± 0.522^b^	7.04 ± 0.566^b^
¼ MS-AC	0.375 ± 0.001 ^a^	0.126 ± 0.001^a^	0.50 ± 0.010^a^	163.84 ± 0.537^a^	120.5 ± 0.500^a^	59.24 ± 0.522^a^	7.96 ± 0.566^ab^

Different letters in each column indicate significant differences at *P* ≤ 0.05

### Acclimatization stage

**Fig. 7:**
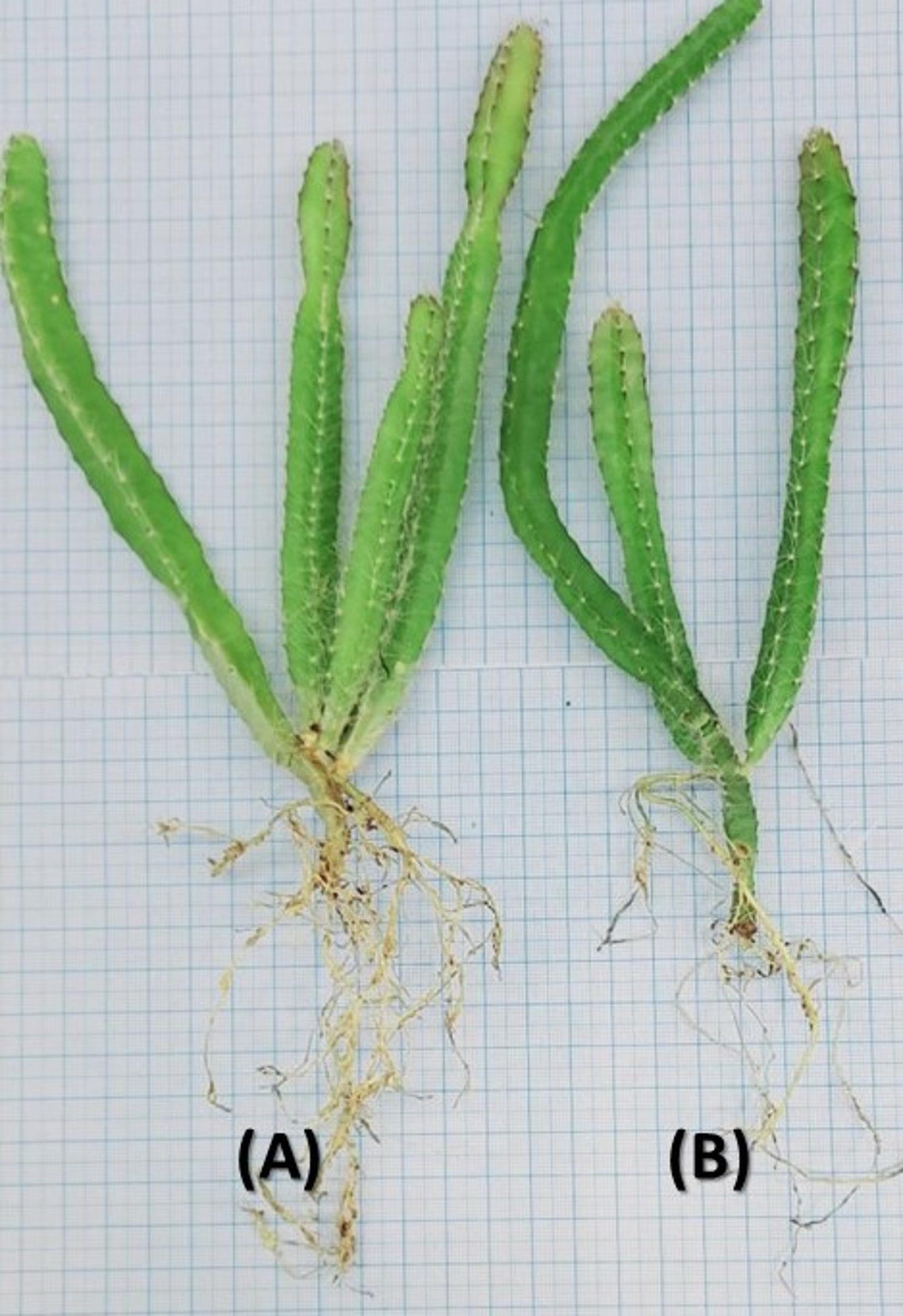
Phenotypes of *S. costaricensis* in response to the Acclimatization culture media (**A**: Peat moss: Perlite: (1:1); **B**: Peat moss: Vermiculite (1:1))

In the acclimatization stage, different soil mixtures were applied for the best growth peat moss: perlite: (1:1) and peat moss: vermiculite (1:1). The medium mixture for hardening significantly affected the measured morphological and physiological parameters (Tables 10 and 11 respectively). Based on the results, the best treatment for shooting and rooting was peat moss: perlite: (1:1), which resulted in the highest plantlet survival (97%), shoot number (3.6), root number (10), root length (23 cm), and plantlet fresh weight (33.1 g). Furthermore, an improvement in all measured physiological parameters was detected in response to the use of peat moss: perlite: (1:1). Dragon plantlets acclimatized with perlite mixture exhibited more robust growth compared to those acclimatized with the peat moss: vermiculite mixture. Figure 7 shows the phenotypes of acclimatized dragon plantlets after 25 days, suggesting a perlite mixture for hardening of plantlets.

**Table 10:** Effect of medium mixture on plantlet survival percentage, plantlet height, shoot number, root length, root number and plantlet fresh weight/ plantlet during *ex vitro* acclimatization stage of *S. costaricensis*

Medium mixture (v/v)	Plantlet survival (%)	Plantlet height (cm)	Shoot number/ plantlet	Root number/ plantlet	Root length (cm)	Plantlet FW (g)
Peat moss: Perlite: (1:1)	97 ± 1.000^a^	22.633 ± 0.551^b^	3.667 ± 0.577^a^	10 ± 1.000^a^	23.833 ± 0.351^a^	33.100 ± 0.265^a^
Peat moss: Vermiculite (1:1)	94 ± 1.000^b^	26.167 ± 0.473^a^	2.333 ± 0.577^b^	6 ± 1.000^b^	16.033 ± 0.153^b^	20.200 ± 0. 436^b^

Different letters in each column indicate significant differences at *P* ≤ 0.05

**Table 11:** Effect of medium mixture on physiological parameters during *ex vitro* acclimatization stage of *S. costaricensis*

Treatment	Chl a + b (mg/g FW)	Carotenoids (mg/g FW)	Total pigments (mg/g FW)	Carbohydrates (mg/g DW)	Protein (mg/g DW)	Total Phenolics (mg/g DW)	Flavonoids (mg/g DW)
Peat moss: Perlite (1:1)	0.508 ± 0.001^a^	0.18 ± 0.001^b^	0.514 ± 0.015^a^	125.51 ± 0.768^a^	293.5 ± 1.04^a^	73.58 ± 0.799^a^	7.55 ± 0.548^a^
Peat moss: Vermiculite (1:1)	0.369 ± 0.001^b^	0.142 ± 0.001^a^	0.414 ± 0.010^b^	97.46 ± 0.746^b^	257.3 ± 0.681^b^	68.08 ± 1.001^b^	6.81 ± 0.548^b^

Different letters in each column indicate significant differences at *P* ≤ 0.05

## Discussion

The composition and strength of growth media, and nutrient concentration changes significantly impact all stages of plant development. This study aims to identify and establish the best in vitro and *ex vitro* conditions for the thriving of *S. costaricensis* (dragon fruit with red pericarp and red flesh) at different micropropagation stages: establishment, multiplication, rooting, and acclimatization. The study evaluated the effects of two basal media (MS and WPM), different media strengths, sucrose concentrations, and plant growth regulators on tissue culture protocol for plant propagation.

Generally the media type influences plant growth [46], MS medium tends to enhance shoot proliferation and overall biomass more than WPM, especially in herbaceous plants [29, 47–49]. A combination of ¾ MS with 3% sucrose produced the highest plant growth in the establishment stage. Regardless of the media type, higher sucrose concentration (3%) and three-quarters-media strength (¾ MS2 or ¾ WPM) produced better results, possibly due to the growth medium’s lower water potential, which can affect the shoot growth significantly, as reported previously [50]. Reducing the basal salt concentration to three-quarters strength (¾ MS or ¾ WPM) often results in improved morphogenic responses compared to full-strength media. This effect is primarily attributed to the lower ionic strength, which minimizes osmotic stress and nutrient toxicity commonly associated with high-salt formulations such as MS. Several studies have demonstrated that diluted media enhances root elongation, shoot proliferation, and overall physiological balance in sensitive species. For instance [51], reported that half-strength WPM significantly improved root development in *Cistus creticus*, suggesting that reduced salt levels favor water uptake and cell expansion. Similarly [52], emphasized the need to move beyond traditional full-strength MS formulations toward customized media compositions tailored to species-specific requirements. These findings indicate that adjusting macronutrient concentrations to ¾ strength can optimize nutrient availability without compromising growth, thereby improving in vitro performance for many horticultural crops. Moreover, higher sucrose levels increase the energy availability required for growth [53]. Sucrose plays a pivotal role in vitro propagation and acclimatization of *S. costaricensis* by acting as both an energy source and an osmotic regulator. Elevated sucrose concentrations in culture media enhance growth by supplying readily available carbohydrates for respiration and biosynthesis, which is critical under photoheterotrophic conditions where endogenous photosynthesis is limited. This energy availability supports rapid cell division, shoot elongation, and root initiation during the multiplication and rooting phases. Recent studies confirm that optimizing sucrose levels significantly improves morphophysiological traits and acclimatization success [54]. demonstrated that a sucrose concentration of approximately 2.3% combined with moderate light intensity enhanced plantlet survival, shoot length, and stomatal development in kiwifruit, highlighting the importance of sucrose in maintaining water balance and promoting photosynthetic competence during acclimation. Similarly [55], reported that sucrose-enriched media improved shoot proliferation and rooting in *Gymnocalycium* cv. Fancy, resulting in vigorous plants that acclimatized successfully.

In the multiplication stage, MS supplied with lower BAP was better in the cluster formation of shoots and their characteristics. However, increased BAP decreased shoot number and biomass. As the BAP concentration increased, there was a noticeable decline in both the number and quality of developed shoots from dragon fruit explants. Lower concentrations of BAP were more effective in producing healthy plantlets with reduced apical death [23]. These results are consistent with broader reports on Cactaceae micropropagation, where cytokinin overdose often disrupts apical dominance and leads to physiological stress, reducing overall plantlet quality. Therefore, optimizing BAP concentration is critical for achieving balanced shoot proliferation and minimizing apical death during in vitro propagation of *Hylocereus* species [56, 57]. The cladode number proliferated in this study (70.66) was higher than those recorded in prior studies on *H. polyrhizus* [26] and *H. costaricensis* [51, 58]. This suggests the enhanced conditions established during this study contributed to improved growth environment, optimal nutrient uptake, and plant growth regulators balance. This led to a significant improvement in shoot number and biomass.

The study found that ¼MS medium promoted rooting more efficiently than ¼WPM medium, contrasting with a previous study on two pitaya species (*H. undatus* and *H. polyrhizus*) showing WPM medium produced a higher average number of roots and MS gave slightly longer roots [53]. Other studies were in line with our results and showed that MS at low strength significantly enhanced rooting [51], suggesting that lower concentrations may facilitate longer root development.

The best rooting was achieved on ¼ MS media with AC and low IBA concentration. The positive effect of low media strength and IBA on in vitro rooting was also reported for *Artemisia nilagirica* [58]. Application of AC to rooting media improved the root induction and development, with ¼ MS more efficiently than ¼ WPM. This is possibly due to better rooting conditions in dark media, nutrient uptake, and preventing tissue oxidation by adsorbing toxic compounds from media [36]. This results in high-quality roots and enhanced growth along with increased fresh weight [59]. Additionally, AC enhanced the overall plant growth [37]. In this connection, enhanced shoot growth with a significant accumulation of photosynthetic pigments and phytochemicals was demonstrated in plants grown on media with AC (¼ MS-AC and ¼ WPM-AC) compared to media lacking AC.

Different media mixtures affect the in vitro hardening of dragon plants. The combination of peat moss: perlite (1:1) showed promising results for the growth of dragon plantlets during hardening. This medium mixture improved the survival rate, morphological parameters, and physiological characteristics, indicating its effectiveness in promoting overall plant growth. The superiority of perlite over vermiculite in hardening dragon plantlets might be due to its ability to provide adequate aeration and drainage for the roots, promoting healthy growth and development [60]. Additionally, the perlite helped prevent soil compaction, allowing for better root penetration and nutrient uptake.

Overall, the current study shows that improving each step of *S. costaricensis* micropropagation requires careful consideration of baseline media strength, sucrose supply, and plant growth regulators balance. According to earlier research, moderate medium strength and sufficient carbohydrate availability promote cactus tissue growth by enhancing osmotic balance and energy supply. This is supported by the higher performance of ¾-strength MS with 30 g/L sucrose during establishment [8, 53]. In line with findings that high cytokinins can restrict shoot quality or cause vitrification in dragon fruit explants, low BAP concentrations also markedly increased shoot proliferation [23, 29]. Evidence that lower ionic strength promotes root elongation while charcoal adsorbs phenolics and enhances root system architecture is supported by the fact that root induction was highest on ¼ MS supplemented with activated charcoal [32, 36, 58]. Lastly, the high acclimation success in the peat moss: perlite substrate supports earlier findings that highly aerated substrates improve root respiration and water-air balance during the *ex-vitro* transition [60]. Together, these findings present a comprehensive and effective propagation technique that outperforms pitaya species protocols previously documented, providing a dependable foundation for the large-scale production and preservation of elite dragon fruit genotypes.

## Conclusion

Optimizing in vitro conditions can significantly promote plant mass production. The media type selection, concentration, plant growth regulators application, charcoal, and the acclimatization soil mixture, are critical for producing high-quality dragon plantlets. Furthermore, these factors contribute to the optimal growth at each stage depending on the specific needs of the cultivated plant species. From all the stages applied for the in vitro conditions, it was clear that MS media is better than WPM media. Low concentration of BAP in MS media positively influenced shoot formation in dragon fruit explants. Rooting was optimized by adding activated charcoal to the medium. The combination of peat moss and perlite (1:1 ratio) was more effective for hardening plantlets, resulting in superior growth and survival rates. These specific combinations can be recommended for optimal plantlet development in similar studies or applications, ultimately supporting the successful cultivation of dragon plantlets in vitro, thereby ensuring the continued success and advancement of dragon fruit production on a commercial scale.

## Data Availability

The datasets generated during the current study are available from the corresponding author on reasonable request.

## References

[1] Ali MAA, Nasser MA, Abdelhamid AN, Ali IAA, Saudy HS, Hassan KM. Melatonin as a key factor for regulating and relieving abiotic stresses in harmony with phytohormones in horticultural Plants — a review. J Soil Sci Plant Nutr. 2024;24:54–73. 10.1007/s42729-023-01586-9.

[2] Abou El-Nasr MK, Nasser MA, Ebrahim M, Samaan MSF. Alleviating biotic stress of powdery mildew in Mango cv. Keitt by sulfur nanoparticles and assessing their effect on productivity and disease severity. Sci Rep. 2025;15:5537. 10.1038/s41598-025-88282-z. 39953098 10.1038/s41598-025-88282-z PMC11828861

[3] Abou El-Nasr MK, Hassan KM, Abd-Elhalim BT, Kucher DE, Rebouh NY, Ansabayeva A, et al. The emerging roles of nanoparticles in managing the environmental stressors in horticulture Crops—A review. Plants. 2025;14:2192. 10.3390/plants14142192. 40733428 10.3390/plants14142192 PMC12298009

[4] Ansabayeva A, Makhambetov M, Rebouh NY, Abdelkader M, Saudy HS, Hassan KM, et al. Plant Growth-Promoting microbes for resilient farming systems: mitigating environmental stressors and boosting crops Productivity—A review. Horticulturae. 2025;11:260. 10.3390/horticulturae11030260.

[5] Hassan AH, Mansour N, Samaan MSF, Nasser MA. Improving Naomi Mango trees capability to withstand salt stress using some plant growth regulators. J Soil Sci Plant Nutr. 2025;25:7152–69. 10.1007/s42729-025-02586-7.

[6] Trivellini A, Lucchesini M, Ferrante A, Massa D, Orlando M, Incrocci L, et al. Pitaya, an attractive alternative crop for mediterranean region. Agronomy. 2020;10:1065. 10.3390/agronomy10081065.

[7] Trindade AR, Paiva P, Lacerda V, Marques N, Neto L, Duarte A. Pitaya as a new alternative crop for Iberian peninsula: biology and edaphoclimatic requirements. Plants. 2023;12:3212. 10.3390/plants12183212. 37765376 10.3390/plants12183212 PMC10537634

[8] Li P, Ma X, Li Z, Yao H, Lu G, Hu H et al. A review on the advances of Dragon fruit. T. 2024;3. 10.48130/tp-0024-0041

[9] Yadav A, Dhakar MK, Arunachalam A, Jha S, Garg S, Gangwar N, et al. A Review on the Scope of Adoption of Underutilized Climate Smart Dragon Fruit (*Hylocereus spp.*) Cultivation. Appl Fruit Sci. 2024;66:297–309. 10.1007/s10341-023-01006-3.

[10] Al-Qthanin R, Salih AMME, Mohammed A, Alhafidh F, Almoghram SAM, Alshehri GA, Alahmari NH. Assessing the suitability of Pitaya plant varieties for cultivation in the arid climate of Saudi Arabia. Heliyon. 2024;10:e21651. 10.1016/j.heliyon.2023.e21651. 38163115 10.1016/j.heliyon.2023.e21651 PMC10754707

[11] Kishore K. Phenological growth stages of Dragon fruit (*Hylocereus undatus*) according to the extended BBCH-scale. Sci Hort. 2016;213:294–302. 10.1016/j.scienta.2016.10.047.

[12] Singh A, Swami S, Panwar NR, Kumar M, Shukla AK, Rouphael Y, et al. Development changes in the physicochemical composition and mineral profile of Red-Fleshed Dragon fruit grown under Semi-Arid conditions. Agronomy. 2022;12:355. 10.3390/agronomy12020355.

[13] Brar JS, Sharma S, Kaur H, Singh H, Naik EK, Adhikary T. Phytochemical properties, antioxidant potential and fatty acids profiling of three Dragon fruit species grown under sub-tropical climate. Not Bot Horti Agrobo. 2023;51:12993. 10.15835/nbha51312993.

[14] Shah K, Chen J, Chen J, Qin Y. Pitaya Nutrition, Biology, and biotechnology: A review. IJMS. 2023;24:13986. 10.3390/ijms241813986. 37762287 10.3390/ijms241813986 PMC10530492

[15] Prabowo I, Utomo EP, Nurfaizy A, Widodo A, Widjajanto E, Rahadju P. Characteristics and antioxidant activities of anthocyanin fraction in red Dragon fruit peels (*Hylocereus polyrhizus*) extract. Drug Invention Today. 2019;12:670–8.

[16] Vera Cruz DG, Paragas DS, Gutierrez RL, Antonino JP, Morales KS, Dacuycuy EA et al. Characterization and assessment of phytochemical properties of Dragon fruit (*Hylocereus Undatus* and *Hylocereus polyrhizus*) peels. International Journal of Agricultural Technology. 2022;18:1307–1318.

[17] Tristanto NA, Cao W, Chen N, Suryoprabowo S, Soetaredjo FE, Ismadji S, et al. Pectin extracted from red Dragon fruit (*Hylocereus polyrhizus*) Peel and its usage in edible film. Int J Biol Macromol. 2024;276:133804. 10.1016/j.ijbiomac.2024.133804. 38996891 10.1016/j.ijbiomac.2024.133804

[18] Bozkurt T, S¸ims¸ek Ö. Propagation. In: Dragon Fruit. 2024. pp. 93–101. 10.1079/9781800623156.0007

[19] Anushi KA, Ghosh PK. From seed to succulence: mastering Dragon fruit propagation techniques. J Plant Biota. 2024. 10.51470/JPB.2024.3.1.08. 3:2024.

[20] Dirga KT, Maryuandini U, Sachio S, Sukma D. Optimizing tissue culture for yellow Dragon fruit (*Selenicereus megalanthus*) propagation: enhancing shoot and root induction. J Trop Crop Sci. 2025;12:663–71. 10.29244/jtcs.12.03.663-671.

[21] Mežaka I, Kļaviņa D, Kaļāne L, Kronberga A. Large-Scale *In vitro* propagation and *Ex vitro* adaptation of the endangered medicinal plant *Eryngium maritimum* L. Horticulturae. 2023;9:271. 10.3390/horticulturae9020271.

[22] Bahnasy MI, Abdel Razik AB, Ahmed MF, Nasser MA, Mekiso GT, Ahmed EZ, et al. *Vitro* culture of Aegle Marmelos against media composition stress: molecular Identification, media, and enzyme optimization for higher growth yields. Int J Genomics. 2025;2025:4630425. 10.1155/ijog/4630425. 40260051 10.1155/ijog/4630425 PMC12011463

[23] Viñas M, Fernández-Brenes M, Azofeifa A, Jiménez VM. *In vitro* propagation of purple pitahaya (*Hylocereus costaricensis* [F.A.C. Weber] Britton & Rose) cv. Cebra. *In Vitro* Cell Dev Biol-Plant. 2012;48:469–77. 10.1007/s11627-012-9439-y

[24] Bozkurt T, İnan S, Dündar İ. Micropropagation of different Pitaya varieties. Int J Agricultural Nat Sci. 2020;13:39–46.

[25] Bozkurt T, Inan S, Dundar I, Kozak S. Effect of different plant growth regulators on micropropagation of some Pitaya varieties. JTropLifeScience. 2022;12:183–90. 10.11594/jtls.12.02.04.

[26] Dewir YH, Habib MM, Alaizari AA, Malik JA, Al-Ali AM, Al-Qarawi AA, et al. Promising application of automated liquid culture system and arbuscular mycorrhizal fungi for Large-Scale micropropagation of red Dragon fruit. Plants. 2023;12:1037. 10.3390/plants12051037. 36903898 10.3390/plants12051037 PMC10005386

[27] Kasim DP, Kishore NS, Suneetha P, Rao KB, Kumar MN, Krishna MSR. Multiple shoot regeneration in Seed-derived immature leaflet explants of red Dragon fruit (*Hylocereus costaricensis)*. Rese Jour Pharm Technol. 2019;12:1491. 10.5958/0974-360X.2019.00246.4.

[28] Tawfik E, Ahmed MF, Albalawi DA, Aljuaid BS, Darwish DBE, Mahmoud SF, et al. Molecular identification of Zantedeschia culture with determination of its morphometric and metabolic activities for mediterranean acclimatization. Plants. 2022;11:2311. 10.3390/plants11172311. 36079693 10.3390/plants11172311 PMC9460599

[29] Oo KT, Lynn ZM, Oo KZ, Htwe MY, Htet WT, Soe W, et al. *In vitro* propagation of three Pitaya varieties (*Hylocereus undatus*, *Hylocereus polyrhizus* and *Hylocereus megalanthus*) with the use of different BAP concentrations. J Sci Innovative Res. 2023;12:33–9. 10.31254/jsir.2023.12203.

[30] Bayhan N, Yücesan B. The impact of sucrose and 6-benzylaminopurine on shoot propagation and vitrification in *Aronia melanocarpa* (black chokeberry). Plant Cell Tiss Organ Cult. 2024;156:55. 10.1007/s11240-023-02652-x.

[31] Baye E, Matewos T, Belew D. Effect of 6-Benzyl amino purine on *In vitro* multiplication of tomato (*Lycopersicon esculentum* Mill.) varieties using shoot explant. J Plant Sci Agricultural Res. 2020;4:1–9.

[32] Martins JPR, Mokhtari AM, Wawrzyniak MK. Cytokinins combined with activated charcoal do not impair *in vitro* rooting in Quercus Robur L.: insights from morphophysiological and hormonal analyses. BMC Plant Biol. 2025;25:1005. 10.1186/s12870-025-07064-x. 40750842 10.1186/s12870-025-07064-x PMC12315455

[33] Wang T, Li H, Zhao J, Huang J, Zhong Y, Xu Z, et al. Exploration of suitable conditions for shoot proliferation and rooting of *Quercus Robur* L. in plant tissue culture technology. Life. 2025;15:348. 10.3390/life15030348. 40141693 10.3390/life15030348 PMC11943399

[34] Yaman M, Palaz EB, Isak MA, Demirel S, İzgü T, Adalı S, et al. Integrating *In vitro* propagation and machine learning modeling for efficient shoot and root development in *Aronia melanocarpa*. Horticulturae. 2025;11:886. 10.3390/horticulturae11080886.

[35] Da Silva FR, Stefanello CA, de Fraga HP. 6-benzylaminopurine promotes the shoots formation during plantlets *in vitro* culture and affects the photosynthetic pigments accumulation in acclimatized plants of *Maxillaria picta* (Orchidaceae). Plant Cell Tiss Organ Cult. 2025;160:58. 10.1007/s11240-025-03002-9.

[36] Martins JPR, Wawrzyniak MK, Kalemba EM, Ley-López JM, Lira JMS, Chmielarz P. *In vitro* rooting of *Quercus robur*, activated charcoal vs. exogenous auxin: a morphophysiological approach. Plant Cell Tiss Organ Cult. 2024;156:24. 10.1007/s11240-023-02656-7.

[37] Ebrahimi T, Piri K, Abdoli A, Tohidfar M. Effect of activated charcoal on *in vitro* propagation of *Lythrum salicaria* L. Lythraceae). JMPB. 2023; Online First. 10.22034/jmpb.2023.362219.1554

[38] Murashige T, Skoog F. A revised medium for rapid growth and bio assays with tobacco tissue cultures. Physiol Plant. 1962;15:473–97. 10.1111/j.1399-3054.1962.tb08052.x.

[39] McCown BH, Lloyd G. Woody plant medium (WPM)-a mineral nutrient formulation for microculture for Woody plant species. Hort Sci. 1981;16:453.

[40] Metzner H, Rau H, Senger H. Untersuchungen Zur synchronisierbarkeit einzelner Pigmentmangel-Mutanten von chlorella. Planta. 1965;65:186–94. 10.1007/BF00384998.

[41] Umbreit WW, Burris RH, Stauffer JF. Manometric techniques: a manual describing methods applicable to the study of tissue metabolism. Burgess Publ. 1957:338.

[42] Bradford MM. A rapid and sensitive method for the quantitation of microgram quantities of protein utilizing the principle of protein-dye binding. Anal Biochem. 1976;72:248–54. 10.1016/0003-2697(76)90527-3. 942051 10.1016/0003-2697(76)90527-3

[43] Kujala TS, Loponen JM, Klika KD, Pihlaja K. Phenolics and betacyanins in red beetroot (*Beta vulgaris*) root: distribution and effect of cold storage on the content of total phenolics and three individual compounds. J Agric Food Chem. 2000;48:5338–42. 10.1021/jf000523q. 11087483 10.1021/jf000523q

[44] Zhishen J, Mengcheng T, Jianming W. The determination of flavonoid contents in mulberry and their scavenging effects on superoxide radicals. Food Chem. 1999;64:555–9. 10.1016/S0308-8146(98)00102-2.

[45] Zou Y, Lu Y, Wei D. Antioxidant activity of a Flavonoid-Rich extract of *Hypericum perforatum* L. *in vitro*. J Agric Food Chem. 2004;52:5032–9. 10.1021/jf049571r. 15291471 10.1021/jf049571r

[46] Rudiyanto PA, Efendi D, Ermayanti TM. Growth response of four accessions of Moringa Oleifera Linn shoots cultured on various basic media. IOP Conf Ser: Earth Environ Sci. 2021;741:012054. 10.1088/1755-1315/741/1/012054.

[47] Fan Q-J, Zheng S-C, Yan F-X, Zhang B-X, Qiao G, Wen X-P. Efficient regeneration of Dragon fruit (*Hylocereus undatus*) and an assessment of the genetic fidelity of *in vitro*-derived plants using ISSR markers. J Hortic Sci Biotechnol. 2013;88:631–7. 10.1080/14620316.2013.11513017.

[48] Jessica JJA, Nurul Fitriyani M, Noorasmah S, Sreeramanan S. Establishment of *in vitro* of plantlets production of white Dragon fruit (*Hylocereus undatus*) using nodal segments. Trans Malaysian Soc Plant Physiol. 2021;28:222–8.

[49] Li H, Wang H, Guan L, Li Z, Wang H, Luo J. Optimization of High-Efficiency tissue culture regeneration systems in Gray Poplar. Life. 2023;13:1896. 10.3390/life13091896. 37763300 10.3390/life13091896 PMC10532866

[50] Salem J, Hassanein A, El-Wakil DA, Loutfy N. Interaction between growth regulators controls *In vitro* shoot multiplication in paulownia and selection of NaCl-Tolerant variants. Plants. 2022;11:498. 10.3390/plants11040498. 35214831 10.3390/plants11040498 PMC8878327

[51] Ioannidis K, Koropouli P. Effects of different media and their strengths in *In vitro* culture of three different *Cistus creticus* L. Populations and their genetic assessment using simple sequence repeat molecular markers. Horticulturae. 2024;10:104. 10.3390/horticulturae10010104.

[52] Pasternak T, Steinmacher D. Plant tissue culture *In Vitro*: A long journey with lingering challenges. Int J Plant Biology. 2025;16:97. 10.3390/ijpb16030097.

[53] Comlekcioglu S. *In vitro* propagation and rooting strategies for *Hylocereus Undatus* and *Hylocereus polyrhizus*. Plant Biotechnol Rep. 2024;18:829–38. 10.1007/s11816-024-00935-z.

[54] Gago J, Martínez-Núñez L, Landín M, Flexas J, Gallego PP. Modeling the effects of light and sucrose on in vitro propagated plants: A multiscale system analysis using artificial intelligence technology. PLoS ONE. 2014;9:e85989. 10.1371/journal.pone.0085989. 24465829 10.1371/journal.pone.0085989 PMC3896442

[55] Cortés-Olmos C, Guerra-Sandoval VM, Blanca-Giménez V, Rodríguez-Burruezo A. Micropropagation and acclimatization of gymnocalycium cv. Fancy (Cactaceae): developmental responses to different explant types and hormone conditions. Plants. 2023;12:3932. 10.3390/plants12233932. 38068570 10.3390/plants12233932 PMC10708245

[56] Bhanwariya M, Maji S, Meena SK, Chouhan T, Jat K. Effect of various concentrations of n-6-benzyl aminopurine (BAP) on the growth of shoots in Dragon fruit explants. Pharma Innov. 2023;12:476–8.

[57] Comlekcioglu S. In vitro propagation and rooting strategies for Hylocereus Undatus and Hylocereus polyrhizus. Plant Biotechnol Rep. 2024;18:829–38. 10.1007/s11816-024-00935-z.

[58] Shinde S, Sebastian JK, Jain JR, Hanamanthagouda MS, Murthy HN. Efficient *in vitro* propagation of *Artemisia Nilagirica* var. Nilagirica (Indian wormwood) and assessment of genetic fidelity of micropropagated plants. Physiol Mol Biol Plants. 2016;22:595–603. 10.1007/s12298-016-0379-6. 27924132 10.1007/s12298-016-0379-6 PMC5120036

[59] Zahra AF, Ilham EQ, Nagla A, Mohammed I, Rabea Z, Najiba B. Effect of medium Culture, pH, activated Carbon, and light on Germination, rooting and growth of tomato (*Lycopersicum esculentum* Mill). Ujar. 2023;11:389–402. 10.13189/Ujar.2023.110216.

[60] Sharaf-Eldin M, El-Aidy F, Hasssan N, Masoud A, El-Khateeb E. Comparison between soil and soilless cultivation of autumn tomato production under Spanish Net-House conditions. EgyptJ Soil Sci. 2023;63:0–0. 10.21608/ejss.2023.225734.1627.

